# Sequence-based *in silico* analysis of well studied Hepatitis C Virus epitopes and their variants in other genotypes (particularly genotype 5a) against South African human leukocyte antigen backgrounds

**DOI:** 10.1186/1471-2172-13-67

**Published:** 2012-12-10

**Authors:** Nishi Prabdial-Sing, Adrian J Puren, Sheila M Bowyer

**Affiliations:** 1 Specialized Molecular Diagnostics, Hepatitis Unit, National Institute for Communicable Diseases (NICD), National Health Laboratory Services (NHLS), Johannesburg, South Africa; 2 Division of Virology and Communicable Diseases Surveillance, School of Pathology, University of Witwatersrand, Johannesburg, South Africa; 3 Department of Medical Virology, University of Pretoria, Pretoria, South Africa; 4 Tshwane Academic Division, NHLS, Pretoria, South Africa

**Keywords:** Epitope vaccines, Viral variation, Population coverage, HCV, HLA, Epitope prediction

## Abstract

**Background:**

Host genetics influence the outcome of HCV disease. HCV is also highly mutable and escapes host immunity. HCV genotypes are geographically distributed and HCV subtypes have been shown to have distinct repertoires of HLA-restricted viral epitopes which explains the lack of cross protection across genotypes observed in some studies. Despite this, immune databases and putative epitope vaccines concentrate almost exclusively on HCV genotype 1 class I-epitopes restricted by the HLA-A*02 allele. While both genotype and allele predominate in developed countries, we hypothesise that HCV variation and population genetics will affect the efficacy of proposed epitope vaccines in South Africa. This *in silico* study investigates HCV viral variability within well-studied epitopes identified in genotype 1 and uses algorithms to predict the immunogenicity of their variants from other less studied genotypes and thus rate the most promising vaccine candidates for the South African population. Six class I- and seven class II- restricted epitope sequences within the core, NS3, NS4B and NS5B regions were compared across the six HCV genotypes using local genotype 5a sequence data together with global data. Common HLA alleles in the South African population are A30:01, A02:01, B58:02, B07:02; DRB1*13:01 and DRB1*03:01. Epitope binding to 13 class I- and 8 class –II alleles were described using web-based prediction servers, Immune Epitope Database, (IEDB) and Propred. Online population coverage tools were used to assess vaccine efficacy.

**Results:**

Despite the homogeneity of genotype 1 and genotype 5 over the epitopes, there was limited promiscuity to local HLA-alleles.Host differences will make a putative vaccine less effective in South Africa. Of the 6 well-characterized class I- epitopes, only 2 class I- epitopes were promiscuous and 3 of the 7 class-II epitopes were better conserved and promiscuous. By fine tuning the putative vaccine using an optimal cocktail of genotype 1 and 5a epitopes and local HLA data, the coverage was raised from 65.85% to 91.87% in South African Blacks.

**Conclusion:**

While i*n vivo* and *in vitro* studies are needed to confirm immunogenic epitopes, *in silico* HCV epitope vaccine design which takes into account HCV variation and host allele frequency will maximize population coverage in different ethnic groups.

## Background

As a relatively “new” virus, only identified in 1989 [1] and first cultured successfully in 2005 [2], there is still much that is unknown about the hepatitis C virus (HCV) and this has hindered the development of an effective vaccine. The following are some of the challenges to successful HCV vaccine design.

1) The virus is highly mutable and exists as a quasispecies within the host and genotypes cluster geographically.

2) Host cell responses to HCV infection are poorly defined and inconsistent among infected individuals.CD4+ and CD8+ T-cell responses are also not cross-protective to heterologous genotypes [3] and, to date, there is no immunodominant epitope that is consistently found in HCV-positive individuals [4].

3) Humans are the only natural host of HCV, and suitable laboratory models have only been developed recently. The chimpanzee has been infected in the laboratory [5], but studies using this model are expensive and limited. The mouse model for viral pathogenesis studies promises a more practical and plausible alternative [6,7].

Epitope-based vaccines promote an immune response by presenting immunogenic peptides (viral genotype-specific) bound to major histocompatibility (MHC) molecules (host specific) to the T cell receptor. Class II- proteins are presented to T helper cells by antigen presenting cells (APCs) with the aid of the CD4 co-receptor whereas class I- proteins are presented by the infected target cell to cytotoxic T cells with the aid of the CD8 co-receptor. The T helper response is important in directing and activating the immune response, including the effectiveness of CD8+ T cells [8].An effective vaccine must be capable of inducing and maintaining powerful CD4 and CD8 T-cell immunity in the greatest proportion of its target population.

Both HCV genotype and HLA allele frequency are distributed geographically. Viral genotype, host genetic background [9] and HLA class I- [10] and class II- alleles [11] are associated with both HCV disease progression and sustained response to therapy [12]. South Africa has diverse ethnic groups, hence a high diversity of HLA genetic background [13]. Black Africans, including the well-studied Zulu ethnic group, constitute the majority (79.4%) population in the country (Statistics South Africa, [14], http://www.statssa.gov.za/PublicationsHTML/P03022010/html/P03022010.html). Other major population groups include Caucasians (Europeans and Indian/Asian,11.8%) and those of mixed race (8.8%). The predominant HCV genotype in South Africa is genotype 5a. This little studied genotype accounts for 57% of the HCV infections in South Africa with the very well studied genotype 1 accounting for 23% [15]. In comparison, genotype 1 accounts for 70% of HCV infections in USA [16]. Hence, most peptide-based vaccines studies concentrate mainly on HCV genotype 1 epitopes restricted by HLA-A*02 which is the most common HLA allele in populations of European/Caucasian descent (New allele Frequency Database [17], http://www.allefrequencies.net).

The binding of the epitope to the HLA-molecule is a highly selective process as only 1 in 40–200 peptides would bind to the HLA class I- or II- allele with high affinity to produce an efficient immune response [18]. Computer prediction servers have made it possible to identify potentially strong peptide binders to HLA molecules that can then be tested *in vitro* and *in vivo* as putative epitopes for peptide-based vaccines. This is a cost- and time-saving exercise as it is expensive and laborious to synthesize and test several 9-mer or overlapping peptides over long target antigens. There are various computational prediction servers available and their sensitivity is constantly improving, including more than 20 prediction servers to identify HLA-II binding peptides [19].

We hypothesize that putative vaccines based on restriction by the HLA-A*02 allele and genotype 1 sequences will not perform optimally in South Africa. The aim of the study was, therefore, to investigate the heterogeneity of well studied HCV epitope sequences across HCV genotypes (with particular reference to genotype 5a) and assess their immunogenicity against prevalent local HLA-types in order to assess vaccine efficacy and population coverage in the ethnically diverse South African population. This descriptive study used web-accessible prediction servers to predict epitope binding of recently published putative epitopes for HCV vaccines against the South African HLA background. The main objectives of the study were:

1) To characterise the variation of selected published immunogenic epitopes within popular target antigens, focusing on South African genotype 5a data.

2) To predict the immunogenicity of these epitopes and their variants against the background of prevalent alleles in the South African target population.

## Results

### Degree of conservation between epitopes

The Weblogo consensus was generated from individual alignments of all available sequence data of HCV genotypes (1a, 1b, 2, 3, 4, 5a and 6). Thus, seven web logos were generated for each of the 13 chosen class I- (N=6) and class II- (N=7) epitopes (Table 1). The epitopes chosen for this study are well characterized and referenced (Table 1). NS4B^2422-2433^ has only one reference (others have 22–78 references) but it is also the only one that has a different restriction allele i.e. B15. The HCV consensus was derived from the 7 generated weblogos and the percentage conservation within each genotype over the epitope region was calculated as described in the Methods (Table 2 and Additional file 1: Figure S1).

**Table 1:** Six well studied HLA class I- and seven class II- restricted HCV immunodominant epitope sequences were chosen from previous publications for this study

**CLASS I EPITOPES**	**SEQUENCE (Subtype)**	**RESTRICTION**	**REFERENCE**	**NUMBER OF REFERENCES LISTED AT IEDB**
NS3 1073-1081	CINGVCWTV (1a)	A02	[20,21]	78
NS3 1406-1415	KLVALGINAV (1a)	A02	[22,23]	70
NS4 1807-1816	LLFNILGGWV (1a)	A02	[24,25]	39
NS4 1851-1859	ILAGYGAGV (1)	A02	[22]	29
NS5B 2422-2433	MSYSWTGALVTP (1)	B15	[22]	1
NS5B 2727-2735	GLQDCTMLV (1)	A02	[22]	22
**CLASS II**				
Core 17-35^	RRPQDVKFPGGGQIVGGVY (1)	Undetermined Class II allele	[26]	1
Core 21-40^	DVKFPGGGQIVGGVYLLPRR (1)	HLA-DRB1*1501	[21,26,27]	13
NS3 1248-1261	GYKVLVLNPSVAAT (1)	HLA-DRB1*1201; 1101; 1301; 0401	[21,25,28]	5
NS4A 1781-1800	LPGNPAIASLMAFTAAVTSP (1a)	Undetermined Class II allele	[25]	3
NS4A 1801-1820	LTTSQTLLFNILGGWVAAQL (1a)	Undetermined Class II allele	[25,27,29]	4
NS5 2571-2590	KGGRKPARLIVFPDLGVRVC (1a)	Undetermined Class II allele	[4,25,27,29]	4
NS5 2661-2680	QCCDLDPQARVAIKSLTERL (1a)	Undetermined Class II allele	[27,29]	4

^Class II- restricted epitopes in the core region are overlapping sequences.

**Table 2:** The sequences of the chosen epitopes were compared to the consensus sequence and conservation scores (as percentages) were calculated

**CLASS I EPITOPE**	**Consensus Epitope sequence**	***HCV GENOTYPES***						**Mean across genotypes**	**MAX.**	**MIN.**	**SD**	**p-value**
		**1**	**2**	**3**	**4**	**5**	**6**					
NS3^1073-1081^	CINGVMWTV	78	67	67	67	78	67	70.67	67	78	5.680	0.3062
NS3^1406-1415^	LTSLGLNAV	67	56	67	78	67	56	65.17	56	78	8.280	0.1645
NS4^1807-1816^	LLFNILGGW	100	78	78	100	100	78	89.00	78	100	12.049	0.6513
NS4^1851-1859^	ILAGYGAGV	89	67	89	78	89	67	79.83	67	89	10.815	0.2231
NS5B^2422-2433^	MSYSWTGAL	89	89	89	100	89	67	87.17	67	100	10.815	0.406
NS5B^2727-2735^	GLRDCTMLV	78	56	44	78	78	33	61.17	33	78	19.823	0.4142
	Mean within genotypes	83.50	68.83	72.33	83.50	83.50	61.33					
**CLASS II EPITOPE**	**Consensus Epitope sequence**	**1**	**2**	**3**	**4**	**5**	**6**	**Mean across genotypes**	**MAX.**	**MIN.**	**SD**	**p-value**
CORE^17-40^	RRPQDVKFPGGGQIVGGVYLLPRR	100	96	66	96	96	96	91.67	67	78	5.680	0.3062
NS3^1248-1261^	GYKVLVLNPSVAAT	100	93	93	100	100	93	96.50	93	100	3.834	0.32
NS3^1781-1800^	LPGNPAVASLMATAAVTSP	85	80	95	85	90	65	83.33	65	95	10.327	0.4142
NS4^1801-1820^	LTTSQTLLFNILGGWVASQL	85	65	80	90	85	70	79.17	65	90	9.703	0.962
NS5B^2571-2590^	KGGRKPALIVYPDLGVRVC	80	80	90	95	95	80	86.67	80	95	7.527	0.2231
NS5B^2661-2680^	QCCDLEPEARVAIKSLTERL	85	55	70	80	60	50	66.67	50	85	14.023	0.4159
	Mean within genotypes	89.17	78.17	82.33	91.00	87.67	75.67					

The comparative variability of the epitope sequences within and across the different genotypes is shown in Table 2. Genotypes 2 and 6 have the lowest mean intra-genotype scores for both class I- and II- epitope sequences, indicating a greater variation among subtypes within these genotypes. There is only one subtype within genotype 5 so not surprisingly the epitope sequences, including our sequences, from subtype 5a are relatively conserved. Because a large proportion of sequences on the database belong to genotype 1a or 1b, the consensus sequences that were generated is mostly representative of genotype 1 sequences. Mean conservation scores of genotype 5 sequences are the same as that of genotype 1 for class I- (both had an average score of 83.5%) and similar for class II- (87.67% versus 89.17%, for genotypes 5 and 1, respectively for the class II epitopes). The intra-genotype variation was not statistically significant for any of the epitopes selected. Two class I- epitopes (NS4B^1807-1816^ and NS5B^2422-2433^) and four of the six class II-epitopes had the highest average conservation scores of more than 80% (Table 2). Published class II-restricted epitopes were, in general, better conserved than the class I- epitopes, both within and across the genotypes (Table 2).Some epitopes were well conserved (NS4B^1807-1816^ and NS5B^2422-2433^) while others (NS5B^2727-2735^ and NS5B^2661-2680^) were highly variable (Table 2).

Most epitopes were identified using genotype 1a sequences, hence it follows that the epitope sequences had greater identity with genotype 1. Genotype 4 epitope sequences showed a consistently high degree of correspondence with the consensus but since this genotype was represented by the smallest data set, this may not be a true reflection of variation within the genotype. Genotype 6 showed the most variability, with a mean conservation score of 61.33% within this genotype, which is to be expected since this genotype is known to be highly variable (Table 2).

### Major HLA alleles

The most common HLA-A, -B and –C alleles in the South African Black population are classified into supertypes as described by [30]. For example, and as seen in Table 3, the A02 supertype includes the A*02:01 and A*68:02 alleles. The A*30:01 allele belongs to the supertype A01A03. This study predicted binding to 13 HLA class I- alleles in 8 supertypes and 8 class II- HLA-DR alleles predominant in the South African population.

**Table 3:** Binding affinity scores of published epitopes and their variants were determined by the IEDB prediction program to relevant supertypes in South Africa

**Gene**	**Epitope sequence**	**Genotype of epitope**		**Class A- Alleles**						**Class B- Alleles**						
			**Supertypes**	**A01**	**A02**		**A24**	**A01A03**	**A01A24**	**B07**			**B58**		**B27**	
			**Allele type**	A*01:01	A*02:01	A*68:02	A*23:01	A*30:01	A*29:02	B*07:02	B*35:01	B*53:01	B*57:01	B*58:01	B*15:03	B*27:05
NS3 (A*02)	CINGVCWTV	1a		17802	*67*	*61*	14908	15501	12611	23637	20927	25523	19827	13679	19257	23485
1073-1081	CVNGVCWTV	1b		16997	*110*	**20**	12228	13122	11766	21885	15696	13382	18288	12132	20367	23007
	SISGVLWTV	2a variant		18961	**11**	**16**	21483	11417	11417	22455	22186	29702	18590	15055	15691	20667
	TVGGVMWTV	3a		19940	*64*	**8**	12677	14750	9776	20729	21877	24623	16182	18054	26500	24303
	AVNGVMWTV	4a variant		17734	**23**	**14**	24001	4015^#^	12036	10753	20258	20595	17093	12996	13641	18882
	CINGVLWTV	5a		15172	**26**	**39**	17548	13613	13865	23524	21854	15854	18628	11203	17516	21090
	CINGVMWTL	5a variant		17922	*140*	*101*	10449	14413	11435	18947	13165	11237	2239	13165	13572	19956
NS3 (A*02)	KLVALGINA	1a		22719	*273*	15048	32261	1830	18800	24242	25216	37253	23529	20557	4839	19019
1406-1415	KLSGLGLNA	1b		19133	*475*	21824	33559	2557	13152	20740	27147	37083	23891	19220	8973	18099
	QLTSLGLNA	4a		20013	7051	15292	33674	12859	12517	26454	24440	37244	22168	26218	7165	19904
	KLVALGINAV	1a		37929	*52*	8564	39134	NO VALUE	31977	19547	42247	34339	NO VALUE	NO VALUE	NO VALUE	26021
	LTGLGINAV	5a		12100	5692	*304*	32426	10980	20519	21309	20981	33652	25012	21599	12577	26332
	QLTGLGINA	5a variant		22408	6972	7419	34672	13389	17488	26117	23541	36968	25569	22283	15466	20054
NS4B (A*02)	LLFNILGGW	1a, 1b, 4, 5a		22942	14359	17095	18086	17906	9175	24903	19854	17154	956^#^	962^#^	5918	23118
1807-1816	MFFNILGGWV	3a		24613	23482	19706	*343*	15640	1707^#^	21757	11817	8151	10769	1251	13832	26621
	LLFNILGGWV	1a, 1b, 4, 5a		32231	**44**	1159^#^	38969	NO VALUE	19453	32445	40287	25767	NO VALUE	NO VALUE	NO VALUE	25868
NS4B (A*02)	ILAGYGAGV	1a, 1b, 5a		20500	**15**	530^#^	30882	15492	10120	11883	21134	37213	22934	20702	3735	20143
1851-1859	ILAGYGTGV	5a variant		20351	**18**	*193*	32028	17493	12563	11272	21994	36657	23555	20603	2196	19849
NS5B (B*15)	MSYSWTGAL	1a, 1b, 4		12612	1522	**24**	2924	2372	5457	1530^#^	**50**	8456	10166	523^#^	*80*	16876
2422-2433	MSYTWTGAL	5a		12133	2640	**22**	8602	2141^#^	7606	2515^#^	*58*	9150	10680	787^#^	*144*	17267
	YTWTGALIT	5a variant		15779	3000	13286	33166	13737	1561	18979	3920	27619	22480	17360	6553	18765
NS5B (A*02)	GLQDCTMLV	1a		18371	**8**	5733	11972	13187	6275	20996	27015	35681	25282	22002	10687	17601
2727-2735	KLQDCTMLV	1b		17735	**7**	3878	6160	2071^#^	9527	17308	26776	35038	23310	18296	3587	16634
	KLRDCTLLV	5a		19744	**13**	14912	15150	**10**	5150	2800	27145	36627	21481	20362	1720^#^	18071
	ALRDCTMLV	4a		19976	**19**	4673	19836	**29**	9982	5384	26302	36740	24190	22343	1206^#^	20027

<50 IC_50_nm, bold, high affinity.

>50 IC_50_nm, <500 IC_50_nm, *italic, intermediate affinity*.

>500 IC_50_nm, #, poor affinity.

No value indicates server produced no binding score.

### Epitope binding prediction

The predicted binding values of the published and “newly predicted” epitopes to prevalent local class I-alleles were generated using the IEDB, ANN prediction server (Tables 3 and 4, respectively). Predicted binding values of the published epitopes to local HLA class II- alleles were generated using the prediction server Propred, Quantitative matrix (Table 5).

**Table 4:** Binding affinity scores of “newly predicted” epitopes and their variants were determined by the IEDB prediction program to relevant supertypes in South Africa

**GENE**	**EPITOPE SEQUENCE**	**GENOTYPE OF EPITOPE**		**Class A- Alleles**						**Class B- Alleles**						
			**Supertypes**	**A01**	**A02**		**A24**	**A01A03**	**A01A24**	**B07**			**B58**		**B27**	
			**Allele type**	A*01:01	A*02:01	A*68:02	A*23:01	A*30:01	A*29:02	B*07:02	B*35:01	B*53:01	B*57:01	B*58:01	B*15:03	B*27:05
NS3	LTGPTPLLY	5a, 1b		**15**	23679	24474	24873	4551	**5**	24599	6188	7688	*448*	**28**	1558^#^	22842
	LHGPTPLLY	1a		10396	24884	27469	21381	17350	**10**	26731	12561	6443	21175	9987	*442*	23420
	FLSTATQTF	5a		*165*	15329	17845	3634	1663	1886	15839	**40**	17977	16320	4231	**8**	18662
	IVSTAAQTF	1a		20409	23323	22013	4758	11756	5496	11246	*75*	13372	814^#^	*425*	*55*	22273
	VLSTVTQSF	1b, 2a		18550	13712	17004	4838	16940	5785	15666	988^#^	29052	11492	1654	**26**	21745
	IVSTDTQSF	4a		19885	22289	20020	12440	14943	5300	6080	*151*	8229	4394	973^#^	**47**	24757
	TLAGPKGPV	5a, 6a		23444	2081^#^	**13**	33957	16907	18949	6657	21854	39095	25027	22108	20499	22034
	TLASPRGPV	1b		22044	1451^#^	**8**	32375	13790	18855	2453	21660	39379	25346	22237	6235	22501
	TLASSRGPV	2a		22034	857^#^	**11**	29481	9965	20464	2095	19022	38571	24511	22225	3353	22267
	TLASAKHPA	3a		21914	*413*	**49**	29038	10681	19694	16284	12935	39038	24637	21967	13015	23364
	TIASPKGPV	1a		22885	7397	**7**	34010	15054	20663	7437	19533	38620	25493	22070	16303	24145
	SVIDCNSAV	5a		21948	**30**	**9**	24435	8789	12923	1702	4571	35486	21627	21514	3381	25586
	SVTDCNTCV	1b		21476	*131*	**24**	30991	15202	21169	19345	15846	19349	26045	22021	11521	22609
	SVIDCNVAV	1b, 2a, 6a		21855	**15**	**6**	22019	7833	13308	3218	4376	31399	24317	21463	3412	24232
	SVIDCNTCV	1a		22281	**25**	**13**	23478	14812	17452	17390	13879	20769	25334	21666	7032	23918
	SVIDCNTSV	4a		22543	**18**	**9**	25166	10636	15522	3402	11164	17097	24124	20942	4646	24512
	ITYSTYGKF	1b, 5a, 2a, 2b, 1a, 4a		16829	22979	16133	*124*	9722	*352*	21954	6132	16141	*354*	**43**	**27**	20982
	LTYSTYGKF	3a		14296	22834	13829	*263*	10036	*379*	22076	3345	11660	860^#^	**41**	**31**	20046
	KVLVLNPSV	1a, 1b, 2a, 2b, 4a, 5a, 6a		23587	**50**	6303	27046	**21**	18669	14670	21450	31145	20648	8842	3129^#^	18558
	RAKAPPPSW	5a, 1b, 2a, 6a		25817	25080	27568	8387	*308*	25791	7172	18126	8580	**31**	**11**	596^#^	22382
	RAQAPPPSW	1b, 3a, 1a		24980	24747	27454	22992	6443	24136	6017	14212	3253	**38**	**8**	1675^#^	17482
	KVWLAPPPSW	4a		24000	4927	22172	26746	*170*	16220	18770	9620	39029	21580	20215	12296	20633
	LTSLGVNAV	5a		5815	3795	**42**	33629	6533	20008	16663	13886	27357	24243	18277	3860^#^	24767
	LTSLGLNAV	5a variant		5305	3082	*64*	32917	6065	18186	16615	16431	29952	24579	19519	7004	23118

<50 IC_50_nm, bold, high affinity.

>50 IC_50_nm, <500 IC_50_nm, *italic, intermediate affinity*.

>500 IC_50_nm, #, poor affinity.

**Table 5:** Binding affinity scores (as percentages) of Class II published epitopes and their variants were determined by the ProPred prediction program to common DRB1* alleles prevalent in the South African population

***Epitope:***	***Sequence***	***HCV Genotype specificity***	DRB1*0101	*0102	*0301	*0401	*0701	*1101	*1301	*1501
**Core**^**17–42**^	**RRPQDVKFPGGGQIVGGVYLLPRRGP**	**1, 2, 5 & 3**^**var**^**& 6**^**var**^								
	VYLLPRRGP	1, 2, 4, 5, 6	0.0%	0.0%	18.0%	0.0%	0.0%	16.0%	**48.0%**	18.0%
	VGGVYLLPR	1, 2, 4, 5, 6	0.0%	0.0%	17.0%	0.0%	9.0%	9.0%	10.0%	20.0%
**NS3**^**1248–1261**^	**GYKVLVLNPSVAAT**	**1, 2, 4, 5, 6**								
	LVLNPSVAA	1, 2, 3, 4, 5, 6	**37.0%**	**54.0%**	**36.0%**	**47.0%**	**28.0%**	17.0%	**34.0%**	**39.0%**
	YKVLVLNPS	1, 2, 4, 5, 6	5.0%	0.0%	0.0%	**30.0%**	9.0%	**31.0%**	**27.0%**	17.0%
**NS4B**^**1781–1800**^	**LPGNPAIASLMAFTAAVTSP**	**1a, 4**^**var**^								
	LPGNPAVAS	2,3, 5, 6	0.0%	2.0%	0.0%	4.0%	0.0%	0.0%	9.0%	0.0%
	LPGNPAIAS	1, 4	0.0%	0.7%	15.0%	4.0%	0.0%	2.4%	0.0%	7.0%
	IASLMAFTA	1	7.0%	**23.0%**	0.0%	0.0%	4.0%	0.0%	14.0%	**21.0%**
**NS4B**^**1801–1820**^	**LTTSQTLLFNILGGWVAAQL**	**1a, 1b**^**var**^**,**								
	LFNILGGWV	1, 4, 5	0.0%	0.0%	16.0%	0.0%	**24.0%**	0.0%	16.0%	**28.0%**
	FNILGGWVA	1, 4, 5	**47.0%**	**47.0%**	0.0%	2.0%	16.0%	**28.0%**	16.0%	**31.0%**
	ILGGWVASQ	4, 5	0.0%	0.0%	**28.0%**	0.0%	0.0%	2.4%	8.0%	0.0%
	LGGWVASQI	4, 5	0.0%	0.0%	0.0%	0.0%	**21.0%**	0.0%	13.0%	**21.0%**
**NS5B**^**2571–2590**^	**KGGRKPARLIVFPDLGVRVC**	**1, 2**^**var**^**& 6**^**var**^								
	VFPDLGVRV	1	0.0%	0.0%	**34.0%**	0.0%	0.0%	0.0%	0.0%	0.0%
	VYPDLGVRV	3, 5	0.0%	0.0%	**35.0%**	0.0%	14.0%	0.0%	0.0%	19.0%
	IVYPDLGVR	3, 5	0.0%	0.0%	**28.0%**	0.0%	0.0%	0.0%	7.0%	0.0%
	LIVYPDLGV	3, 5	0.0%	0.0%	0.0%	0.0%	12.0%	0.0%	3.0%	**60.0%**
**NS5B**^**2661–2680**^	**QCCDLDPQARVAIKSLTERL**	**5**^**var**^								
	LAPEARQAI	1b	0.0%	0.0%	8.0%	0.0%	11.0%	0.0%	4.5%	11.0%
	LDPQARVAI	5	0.0%	0.0%	8.0%	0.0%	0.0%	0.0%	0.0%	0.0%
	LQPEARAAI	5^var^	0.0%	0.0%	**22.0%**	0.0%	12.0%	1.0%	**22.0%**	**26.0%**

#### HLA-A and –B class I- restricted binding

Binding predictions of epitopes and their variants for all available HLA alleles prevalent in the South African population are shown in Table 3.Five of the six HLA class I-published epitopes (NS3^1073-1081^, NS3^1406-1415^, NS4B^1807-1816^, NS4B^1851-1859^ and NS5B^2727-2735^) have been reported to be HLA-A*02 restricted (Table 1). Three of the five published HLA-A*02 restricted epitopes bound the A*02:01 allele as expected (Table 3).

Predictions for the different alleles were in agreement regardless of the programme or algorithm used (IEDB ANN, Propred I, SYFPEITHI) with two exceptions, binding of the 9 amino acid epitopes of NS4B^1807-1816^ LLFNILGGWV and the HLA-B*27:05 binding predictions. The original 10 amino acid NS4B^1807-1816^genotype 1 epitope LLFNILGGWV (which is conserved in genotype 1b, 4 and 5a) predicted to bind with high affinity (44.1 IC_50_nM) to HLA-A*02:01. Neither IEDB ANN nor ProPred I predicted binding between this allele and the two possible 9 mer epitopes, LLFNILGGW and LFNILGGWV while SYFPEITHI predicted binding of 18% and 14%, respectively. One of the shortcomings of IEDB ANN is that it can only predict binding peptides that are of the same length as those in the training set. For this reason, all peptides were re-analysed with all the alleles of interest using the “any length” parameter for epitope length. No other changes were observed to binding predictions listed in Table 3 using these parameters.

The second exception observed was the failure of IEDB ANN to predict binding between any of the epitopes (or their variants) and HLA-B*27:05 which SYFPEITHI and/or ProPred I scored. There was no data supporting restriction of these particular peptides by B*27:05 in the IEDB epitopes database. Both SYFPEITHI and ProPred I use peptide motifs and amino acid matrix based prediction. The following scores using x-[R (K)]-x ^(6–9)^ could explain the scoring of these two packages for NS3^1406-1415^epitopes KLVALGINA, KLSGLGINA (21%^ProPredI^ 7%^SYFPEITHI^, respectively) and variants KLQDCTMLV and KLRDCTLLV (32%^ProPredI^ 12%^SYFPEITHI^, respectively). SYFPEITHI uses x-[R]-x ^(5–8)^-[LFYRHK (MI)]. However, one would expect lower predictions for NS5B^2422-2433^ epitopes MSYSWTGAL and MSYTWTGAL (38%^ProPredI^ 12%^SYFPEITHI^) since only the carboxyl anchor is present but this was not the case.

NS3^1073-1081^, NS4B^1851-1859^ and NS5B^2727-2735^ bound with high affinity to A*02:01 allele, regardless of genotypic variation (Table 3). All variants tested for both NS5B^2727-2735^and NS4B^1851-1859^ were predicted to bind the A*02:01 allele with equal strength (<20 IC_50_nM, Table 3). High and intermediate binding affinities over all variants was also observed for NS3^1073-1081^ and NS4B^1851-1859^ with allele A*68:02 (Table 3), of the A02 supertype.

Two of the variants, SISGVLWTV (genotype 2a) and TVGGVMWTV (genotype 3a) had changes from the wild type N (Asparagine) in position 3 but none of the variants had changes in positions 4, 5 and 7. Interestingly, when all possible alanine exchange peptides were placed into IEDB ANN, the output scores reflected the experimental binding changes for all of the alanine exchange peptides with the exception of the total abrogation of signal for substitutions in positions 3, 4 and 5 (data not shown).Of note, while consistent binding was observed across the supertype A02 for all of the variants of the A*02 restricted epitope NS3^1073-1081^, epitopes of genotypes 1, 3a and 5a (variant) were found to be intermediate binders (Table 3).

The genotype 4a and 5a variants of the HLA-A*02 restricted epitope NS5B^2727-2735^displayed some level of promiscuity as these were predicted to bind with high affinity to the A01A03 supertype allele, A*30:01 (29 and 10 IC_50_nM, respectively), while the genotype 1b variant had low affinity with this allele (2071 IC_50_nM) and the original genotype 1a peptide was not predicted to bind at all. The original peptide and one of the two of three variants of the published B*15-restricted NS5B^2422-2433^ epitope displayed intermediate binding IC_50_ nM values of 80 and 144 (Table 3). This epitope showed the highest cross-reactivity across the supertypes with both the original epitope and one of the genotype 5a variants binding very strongly to A*68:02 (supertype A02) and B*35:01 (B7 supertype; Table 3).

Of the 6 class I- epitopes used in this study, only two epitope variants were found to be promiscuous: MSYTWTGAL (supertypes A02, B07, B27) and KLRDCTLLV (A02, A01A03).In a preliminary attempt to identify conserved epitopes showing greater promiscuity across supertypes, strings of epitopes (other than the ones selected from publications for this study) of the NS3 protein were placed into the IEDB server. Table 4 indicates that five of the eight epitopes were predicted to be promiscuous, binding with high (<50 IC_50_nm) and intermediate (<500 IC_50_nm) affinities to two or more supertypes: LTGPTPLLY (A01, A01A24, B58), FLSTATQTF (A01, B07, B58, B27), ITYSTYGKF (A24, A01A24, B58, B27), KVLVLNPSV (A02, A01A03), RAKAPPPSW (A01A03, B58). Of the five epitopes above, three were conserved among genotypes 1, 2, 4 and 5 (Table 4), ITYSTYGKF, KVLVLNPSV and RAKAPPPSW.

#### Class II- alleles

ProPred II was used to predict binding of the longer class II- epitopes. Before calculating the predicted binding, the programme identifies all overlapping nine amino acid peptides within the input polypeptide. A predicted binding score is given as a percentage of the maximum possible binding (i.e. the highest log value achievable by an optimal peptide) with the chosen allele (Table 5). For example, CORE^17-42^, RRPQDVKFPGGGQIVGGVYLLPRRGP, returned two 9-mer peptides, VYLLPRRGP and VGGVYLLPR, which scored similarly for alleles HLA-DRB1*03:01 and HLA-DRB1*15:01 (Table 5). However, in the context of DRB1*13:01, VYLLPRRGP had a much higher percentage binding score (48%) than its flanking sequence VGGVYLLPR (10%). Note that no class II- epitopes were predicted in the first 14 amino acids of CORE^17-42^. The CORE^17-42^ epitope was well conserved across the genotypes (second only to NS3^1248-1261^, Table 2), but was not predicted to bind with HLA-DRB1*01:01, HLA-DRB1*01:02 or HLA-DRB1*04:01 and only VGGVYLLPR was predicted to bind with HLA-DRB1*07:01 (9%, Table 5).

The most promiscuous class II-epitope was also the best conserved epitope, NS3^1248-1261^(Table 2), specifically the region 1252–1260 LVLNPSVAA, bound all eight of the alleles tested and was the only epitope to bind HLA-DRB1*04:01.The allele HLA-DRB1*15:01 was predicted to bind with all but five of the 18 peptides output by the program (Table 5). The highest percentage of optimal binding (60%) was predicted between peptide LIVYPDLGV within NS5B^2571-2590^ and the HLA-DRB1*15:01 allele.This immunogenic epitope is one of three variants common to genotypes 3 and 5.

The NS3^1248-1261^ epitope YKVLVLNPS was well conserved among genotypes and bound to three DRB1* alleles (Table 5). Interestingly, the epitope KVLVLNPSV, also conserved, bound to two class I- supertypes (Table 4). Another epitope that is a class I- and II- binder is FNILGGWVA (Table 3 and Table 5, respectively).

#### Coverage calculations

The predicted binding scores of published epitopes (Tables 3 and 5) were used to estimate population coverage. Selected programme output (which includes a list of the input epitopes) has been supplied as supplementary figures where indicated.

##### IEDB population coverage

The published class I- and II- epitopes had coverage of 65.85% (Additional file 2: Figure S2) in South African Blacks and 81.36% (Additional file 3: Figure S3) in South African Whites. Corresponding figures when calculations included only the class I- epitopes were 41.76% and 52.70%, respectively (results not shown). By choosing predominantly genotypes 1 and 5a epitopes (“best mix”) predicted to be immunogenic in South African Blacks, the combined class I- and II-coverage in Blacks improved to 91.87% (Additional file 4: Figure S4) while coverage improved to 94.77% (Additional file 5: Figure S5) in the South African Whites.

##### Optitope Population Coverage

The Optitope candidate epitopes were proposed whether the chosen population was “North American Europeans” or Europe (geographical) and results showed coverage of 94.28% (Additional file 6: Figure S6). Alternatively, candidate epitopes were sought using the same HCV alignment data and choosing the Zulu ethnic group (the only South African ethnic group available in OptiTope) and coverage of 75.16% was shown (Additional file 7: Figure S7).

##### Optitope Epitopes and IEDB population coverage

Candidate epitopes chosen for “optimal” vaccines for Caucasians and Zulus, respectively, from the OptiTope analyses described above, were then tested using the South African white and black populations. Local population data was placed into the IEDB population coverage web application as before.

Results indicated that South African Blacks had a 72.64% chance of responding to a putative European “optimal” vaccine while the same vaccine provided 90.55% coverage in the population for which it was designed. The putative “optimal” vaccine for Zulus provided coverage of 73.72% in South African Blacks with 90.79% coverage in Europeans (summarized in Additional file 8: Figure S8).

## Discussion

HCV genotypes and host genetics vary geographically and yet proposed epitope vaccines are most often formulated based on genotype 1 peptide sequence data alone and their restriction confined to the alleles found predominantly in the Caucasian population. This study assesses the efficacy of a putative epitope vaccine designed with this typical sequence bias when used in South African populations. The heterogeneity of epitope regions proposed for HCV vaccines was explored together with their predicted binding, and that of their variants, to HLA alleles common in the South Africa population.

There is a need to examine viral variation within known epitopes, and assess the prevalence and immunogenicity of the variants for relevant host alleles within the target population, before choosing epitopes for inclusion in an epitope vaccine. This study, therefore, focused on subtype 1a, 1b and 5a sequences as these were found to predominate in South Africa [15]. This is the first time that South African genotype 5a data is being compared to well- studied epitope data of other genotypes. Genotypes 3 and 4 have also been found in the South African population but genotype 2 is rare and, to date, genotype 6 has not been identified. In order to improve the representation of genotype 5a, all available sequence data was included in the alignments, including sequences from our own studies and those of [31] (Belgium and South Africa) and [32] (France).

There are numerous epitopes meeting the inclusion criteria that could have been chosen for the study but a final subset was chosen so that it included well studied epitopes considered for multi-epitopic [22], therapeutic [21], minigene [25] and DNA polytope [23] vaccines.Genotype 1 is a well-studied genotype and considerably more sequences were available for the genotype 1 alignments. Class I- and II- epitope sequences of genotype 5a were found to be relatively conserved compared to some of the other genotypes, notably genotypes 2, 3 and 6.Genotype 5 is considered to be a relatively conserved genotype as to date, there is only one subtype of genotype 5 (5a), compared to the highly intra-genotypically variable genotype 6 that partitions into 22 different subtypes, 6a-6v, considerably more than any of the other genotypes [33].

There have been several studies which show a lack of cross-protection across the genotypes [34-36]. With regard to the NS3^1073-1081^epitope, an extensively studied epitope, our study has predicted high and intermediate binding of variant sequences to A02 supertype, indicating a level of cross-reactivity for this epitope. The consensus at the position 2 of NS3^1073-1081^ was an isoleucine (I). The only other common amino acid in this anchor position was Valine (V). Valine was conserved at position 9 in all but the genotype 5a sequences where approximately one third of the sequences had a leucine (L) in this position. Despite the fact that substitutions at P2 were conservative (an I or V for the more favourable L), affinity of this epitope was lowered. When alanine exchange peptides were used in *in vitro* assays [37], substitutions at positions 3, 4, 5 and 7 of the published NS3^1073-1081^ epitope abolished IFN-gamma production. Changes at positions 2, 8 and 9 only partially reduced production and only positions 1 and 6 had no effect. Even single amino acid exchanges at non-anchor sites can significantly limit the potential efficacy of a vaccine containing only the wild type peptide [37].

[36] identified distinct polymorphism profiles of genotypes 1a and 3a non-structural gene sequences. Only 2 of the 51 polymorphisms, observed to have significant HLA association, were common to both genotypes [36]. The extent of genetic diversity can result in a distinct repertoire of HLA-restricted viral epitopes for different genotypes. When we looked at consensus alignments of the chosen epitopes, we also observed this phenomenon. The consensus at each site of an epitope represents the amino acid best adapted to T cell responses across the host population [36]. A consequence of this is that escape of a mutant (driven by the selection pressure of dominant HLA alleles within the host population) can become the most dominant amino acid. When this happens, the polymorphism in the epitope, or negatope, as it is now called, is over-represented even in hosts not having the allele which drove the escape [36].

One of the shortcomings of IEDB ANN is that it can only predict binding peptides that are of the same length as those in the training set. Hence, the server will not pick up binding in longer epitopes if this is not specified [38]. However, by using older programs, such as SYFPEITHI and BIMAS that use peptide motifs and amino acid matrix based prediction ([39]; Singh and Raghava 200) both of which are popular, updated and have relevance [40] we were able to flag the longer epitopes and repeat the prediction in IEDB ANN for the 10 amino acid epitope.

Epitopes which are well conserved and show good binding affinities to many HLA alleles (promiscuous) are the best candidates for *in vitro* and/or *in vivo* testing. Epitopes like NS4B^1801-1820^are particularly appealing since they contain substrings which act as class I- and class II- alleles. While *in silico* planning has been found to greatly facilitate peptide design, not all peptides predicted *in silico* are optimally immunogenic *in vivo*[41] and it remains essential to test predicted peptides *in vivo* so as to ascertain that the needed T-cell response is elicited. Numerous *in silico* studies have shown the value of using prediction programs to assess the efficiency of binding of putative epitopes to human alleles [42-45]. Also, [46] showed an increase in the use of *in silico* prediction studies with an improvement of epitope prediction programs available. Of the published epitopes used in this study, only 2 class I- (based on binding to ≥supertypes) and 3 class II- (binding to >2 DRB1* alleles) epitopes were found to be promiscuous using the prediction programs.

The NS3 protein is a large protein and has been shown to generate effective immune responses, which can resolve acute infection. This study looked across the NS3 protein to identify possible additional epitopes (other than the ones chosen from the published papers) that may be good binders to predominant HLA-alleles in the South African population. The results of this search (Table 4) which we have called, “newly predicted” NS3 epitopes were found to be well-conserved and bind to more than one HLA class I- allele. Three class I- epitope sequences were found to be highly conserved, particularly among genotypes 1 and 5, and were predicted to be strong binders to two or more supertypes. None of these “newly predicted” NS3 epitopes were found on the Los Alamos HCV immunology database (http://hcv.lanl.gov/content/immuno/tables/ctl_summary.html, accessed 05-09-2012). This exercise illustrates the usefulness of *in silico* studies to identify potential binders which will suit the target populations. *In vivo* studies will always be needed to confirm immunogenicity of these predicted peptides but this study has shown that *in silico* prediction can consider both host and viral variation, particularly in countries like South Africa and Egypt where genotypes other than genotype 1 predominate. *In silico* coverage calculations can not only identify promiscuous epitopes but also optimise the best cocktail for an effective multi-epitope vaccine. A recent *in silico* study identified 69 promiscuous HCV class I- and 150 class II- epitopes that were predicted to bind to genotype 3a [44]. A string of 18 conserved and promiscuous immunodominant epitopes spanning 8 HIV-1 proteins produced an effective immunogen [47], 23 epitopes were found promiscuous to MHC class I- and II- within *E-coli* 536 genome [45] and 15 promiscuous epitopes were predicted within *M. tuberculosis* peptide [43].

This study focused mainly on A02 –restricted epitopes and promiscuity was poor. However, immunogenic epitopes restricted to other alleles have been identified [48-50]. Two B alleles, B57 and B27, have been found to provide spontaneous control of HCV. Neither of these alleles are prevalent in South African Blacks (Paximadis et al., 2011) but preliminary investigations on NS5B (B*57-restricted) epitope, KSKKTPMGF (genotype 1a, [48]), and genotype 5a variants RSKKTPMAF and KSKKIPMAF showed promiscuity to B*58:01, B*15:03 and A*30:01(data not shown). Indeed, this reiterates the need to look at viral variation and promiscuity as this is particularly important to vaccine design.

The following class I- and II-restricted epitopes were selected from the original epitope set as likely to provide the best vaccine in the South African setting. This was based on binding affinities predicted for epitopes expected in the local population and binding to several supertypes recently recommended for inclusion in a vaccine which is optimal for both White and Black South Africans (supertypes A1, A2, B07, B27 and B58; [13]).

1. NS3^1073-1081^ both wild type genotype 1a CINGVCWTV and genotype 1b CVNGVCWTV because they are so well studied and show cross-reactivity within variants and across the supertype A02.

2. NS4B^1807-1816^ (LLFNILGGWV; [22,24,25]) because the 10-mer peptide is well conserved (genotypes 1a, 1b, 4, 5a) and is immunogenic for both class I- and class II- alleles.

3. NS5B^2422-2433^, both the original MSYSWTGAL (genotypes 1a, 1b and 4; Table 3; [22]) and the genotype 5a variant MSYTWTGAL as they cover the supertypes B27 as well as B07 and are also the best available B58 candidate in the recommended supertype set [13].

4. NS5B^2727-2735^genotype 5a variant KLRDCTLLV of the published epitope sequence GLQDCTMLV [22] as it brings the most prevalent HLA-A allele in the Black population (A*30:01) and the most prevalent HCV genotype 5a in South Africa into the mix.

5. The class II-restricted epitopes NS3^1252-1260^ LVLNPSVAA [27] which is conserved in all genotypes and also very promiscuous.

6. NS4B^1809-1817^ which overlaps class I-restricted 1807 (FNILGGWVA; [25]) and is restricted by the 2 HLA-DR alleles in the Black population (HLA DRB1*13:01 and *11:01) and is also promiscuous.

7. Core class II- epitope VYLLPRRGP (genotypes 1,2,4,5,6) included as it is the most reactive of the class II- epitopes to HLA DRB1*13:01.

The frequencies of the most common HLA alleles in the South African Caucasian and Indian populations closely correlate with values from their respective populations globally. However, the frequencies of the most common HLA-A and –B alleles in the South African Black population are both heterogeneous and unique and quite distinct even from other Black populations in Western and Northern Africa [51]. Many of the well studied published and “newly predicted” epitopes assessed in this study bound to A*68:02 (supertype A02). HLA-A*68:02 was found 2.6x more often in the Black population than HLA-A*68:01 (A03 supertype, [13]).

There is a good correlation between immunogenicity and MHC class I- binding affinity [52]. Based on this principle, several web-based resources are available which can assess the population coverage of putative epitope vaccines based on the predicted binding of the epitopes and their variants to chosen HLA alleles relevant to the population being assessed. The predicted coverage of the original well studied class I- and II-epitopes selected for this study to illustrate the drawbacks of a vaccine using South African host population frequencies was found to be 65.85% and 81.36% for Blacks and Whites, respectively (Additional file 8: Figure S8).The OptiTope example highlighted the fact that the greater the knowledge of local viral variation and the immunogenicity of these variants together with accurate high resolution population allele frequencies allows the design of superior epitope vaccines with much better coverage for more groups within the target population. Fine tuning the vaccine by using an optimal cocktail of genotype 1 and 5a epitopes raised the coverage of the vaccine to 91.87% and 94.77%, close to the 100% coverage predicted by [13] in their study population.

## Conclusion

In light of data generated in this study, epitope-based HCV vaccines should contain a mixture of epitope variants from all of the genotypes as wild-type genotype 1 response is not guaranteed to cross-protect against variants, even if the variant is restricted by the same allele. In addition the efficacy of a proposed epitope vaccine will differ between the major population groups. While coverage estimates can be made based on South African supertypes, cross-reaction of peptides with all supertype members is not universal. Clearly for a set of epitopes to elicit a broad and potent immune response in the target population, viral variation and population genetics data should be factored into the algorithm particularly in the light of less-studied variants such a genotype 5a.

Even where proposed epitopes are conserved, host differences will make the vaccine less effective in the South African setting. Of the 13 published and well-characterised epitopes selected for this analysis (including variants from two of these) four class I- and three class II-restricted epitopes would be beneficial in a multi-topic therapeutic vaccine for genotype 5a infection in our population. Hepatitis C genotypes and high resolution population data is necessary when planning epitope vaccine design. While *in vivo* and *in vitro* studies are needed to confirm predicted immunogenic epitopes, *in silico* “reverse immunology” studies provide a sound basis with which to screen the many possible candidates. This study has shown that with the ease and usefulness of web-based sequence- and structure-based prediction servers, non-bioinformaticians can predict potential binders, without expensive computer hardware and programming knowledge.

## Methods

### Epitope sequences

The literature was searched for known immunogenic class I- and II-restricted epitope vaccine candidates. All of the open reading frames (ORF), from the core to the NS5B protein, yielded putative epitopes and these ranged in length from 9 base pairs (bp; [22]) to 683 bp [53]. Six class I- and seven class II- epitopes were chosen for the analyses (Table 1) based on the following criteria:

1. All were extensively studied immunogenic epitopes (as indicated by the number of references in Table 1).

2. All had been published in the peer reviewed literature.

3. All class I- epitopes had known HLA restriction.

4. All had been recommended for putative vaccines.

5. All were from conserved regions of the genome (core to NS5 region).

Alignments of representative reference sequences were obtained over the chosen putative epitope regions using sequence data from each of the genotypes with the aid of pre-aligned and updated amino acid sequence data from the International Nucleotide Sequence Database Collaboration (INSDC; [54]).

The total number of sequences, available per epitope region, varied in numbers by genotype and region on the genome. Genotype 1 (subtypes 1a and 1b) sequences form by far the major number of sequences on the database ranging from 54% (of the total number of sequences) to 84% in some regions. In contrast, the little studied genotypes, genotype 4 and 5, accounted for only 4 to 24% of available sequences, respectively. Genotype 5a is one of the major genotypes found in South Africa together with genotype 1. Thus, to have this local type adequately represented in the data set, we included our own sequence data (25 patients) from the core [GenBank:JX571010-JX571031], NS4B [GenBank: JX571032-JX571039] and NS5B [GenBank: DQ482799-DQ482824] regions of genotype 5a.Care was taken to ensure that all our own data, as well as data used from public databases, corresponded to one sequence per subject. The study was retrospective and approved by the ethics committee of the University of the Witwatersrand, Johannesburg, South Africa (WITS HREC M051114), and was therefore performed in accordance with the ethical standards of the 1964 Declaration of Helsinki. PCR and sequencing was performed as previously described [15,31].

BioEdit (version 7.0; [55]), was used to align all the amino acid sequences. The consensus sequence of immunogenic regions, for each of the genotypes, was generated using the Web based software package, WebLogo (version 2.8.2; http://weblogo.berkeley.edu/logo.cg; 2008-09-08). Sequence numbering is according to [56]. WebLogo produces a consensus of the input sequences output as a series of “letter stacks”, each representing a single column of the sequence alignment (Additional file 1: Figure S1).The height of each letter within the stack is proportional to the relative frequency of the representative amino acid at that position in the sequence [57]. The Weblogo software incorporates a “small sample number” correction, to correct for potential bias.

The relative conservation of each epitope was calculated as a percentage of the number of polymorphic sites over the epitope length when compared to the overall HCV consensus sequence. The HCV consensus was determined by taking the most common amino acid at each amino acid site of the 7 respective genotype consensus sequences (genotypes 1a, 1b, 2, 3, 4, 5a and 6), irrespective of representation in the database. A minimal class I-restricted epitope length of 9 nucleotides was used for all class I-restricted epitopes. Since class II-restricted epitopes are longer and are made up of numerous overlapping regions, the number of amino acids per epitope varied. The statistical analysis was performed using the analysis of variance (ANOVA) tests of significance in the Statistica software, version 9.1.

### Common South African HLA alleles

Initially, a literature search was conducted in order to collate available South Africa population HLA-A –B and –DR allele frequency data which included relevant data stored online in the New allele Frequency Database (http://www.allelefrequencies.net 2010-11-30). However, much of this data was low resolution with 2 digits. Hence, high resolution data [13], which is required for the predictions, were used for the study.

### Immunogenicity prediction and population coverage calculations

Two servers (Immune Epitope Database, IEDB (http://tools.immuneepitope.org, [58]) and Propred II, http://www.imtech.res.in/raghava/propred/index.html, [59]) were chosen for this study because these were user-friendly, easily available online and displayed many of the HLA alleles prevalent in SA. To predict binding to HLA class1- alleles, the IEDB server was used. The Propred II server was used to predict binding to HLA class II- alleles.

#### Resources of the immune epitope database (IEDB)

The IEDB is a manually curated database of experimentally characterized immune epitopes. Its companion site, the IEDB resource, is a collection of tools for prediction and analysis of immune epitopes (http://tools.immuneepitope.org/main/jsp/menu.jsp; version 2.0, accessed 2009-09-09 to 2011-03-14, [60]). The “Peptide Binding to MHC class I- molecules” resource, which predicts MHC binding to T cell epitopes, was utilised for class I- predictions. Valid input data include proteins or peptides. The programme splits these into all possible overlapping peptides and then predicts their binding to each selected MHC allele using the chosen prediction method. The sequence-based method, using the artificial neural network (ANN) algorithm of [61] on the IEDB server was selected for all HLA class I-predictions as it is reported to be more reliable than earlier matrix algorithms [61].

In addition, however, the matrix-based methods, ProPred 1 (http://www.imtech.res.in/raghava/propredI/index.html, 2010-11-30, [62]) and SYFPEITHI [39] were used in parallel and binding efficiencies of the three methods compared. For brevity, only scores for IEDB are shown in the result tables and incompatible results are discussed where appropriate. ANN uses training data from the IEDB to calculate the affinity of a given peptide for specific MHC molecules. It calculates binding based on the position of each amino acid in the putative epitope while taking into account the probability of adjacent amino acids competing for a space in the MHC pocket. Predicted binding efficiencies are calculated in units of IC_50_nM (the half-maximal inhibitory concentration). IC_50_ values <50 nM indicate high affinity while values >500 but <5000 nM indicate low affinity and values in between the two extremes (>50 nM but <500 nM) indicate intermediate affinity (http://tools.immuneepitope.org/main/jsp/menu.jsp).

Sequence data in the NS3 region that was available on the database was used for the genotype 5 conservation score and binding to predominant HLA-alleles in the South African context were predicted.The promiscuity of “newly predicted” (i.e. other than published epitopes) class I-epitopes of the NS3 gene were analysed using the IEDB server. An epitope sequence that bound with <500 IC_50_nM to more than one HLA class I- allele was considered promiscuous.

#### ProPred MHC class II- binding prediction

A structure-based method with a quantitative matrix (QM) algorithm on the Propred II server (http://www.imtech.res.in/raghava/propred/index.html, 2010-10-20, [63]) was used to predict binding of HLA class II- epitopes. This tool uses a linear prediction model which scores the binding potential of the query peptide based on values stored in allele specific coefficient tables, or quantitative matrices. Matrices are generated based on experimental results taking into account the properties of each individual amino acid and its position within the epitope.

The program is useful in locating promiscuous, versus allele specific, binding regions in a query peptide sequence. Note that, by comparison to IEDB ANN, a high score is indicative of good binding between the relevant peptide and the specific HLA allele and vice versa. The score represents the percentage binding of the query peptide when compared to the highest possible binding score for the optimal peptide with the given allele and thus reflects the binding characteristics of the query peptide. However, there is no clear cut off as with IEDB ANN scoring, and actual percentages should not be compared between alleles. The stringency threshold of the analysis can be set between 1% and 10% where the highest stringency guarantees no false positives and the lowest stringency guarantees no false negatives. The highest stringency was, therefore, used in all programme runs to minimize the number of false positives and ensure that all binding had significance.

#### Population coverage calculations

Population coverage was calculated by the Population coverage tool on the IEDB server (http://tools.immuneepitope.org/tools/population/iedb) for South African Whites and Blacks for both the published class I- and II- epitopes and an adapted “best mix” which took into account the most prevalent alleles and epitope variants in South Africa and their predicted binding. In order to assess the efficacy of a vaccine epitope, the IEDB resource Tool calculates the fraction of individuals predicted to respond to a given set of epitopes with known MHC restrictions (http://tools.immuneepitope.org/main/html/analysis_tools.html last accessed 2011-04-20). The calculation is based on input HLA genotypic frequencies.

Recently released web-based software, OptiTope [64], looks at viral and host variation in order to customise and optimise candidate epitopes to a specific population. Since this approach used the same parameters as this study, it was decided to compare the coverage of the chosen epitopes with the coverage of putative optimal epitope vaccines generated in OptiTope using similar biases. For this reason OptiTope was asked to generate an optimal epitope vaccine from an alignment of “common” HCV sequences in a Caucasian population. This HCV sample data (available in OptiTope), while biased, was very comprehensive and consisted of an alignment of >100 sequences from 10 different HCV proteins (Core, E1, E2, NS2, NS3, NS4A, NS4B, NS5A, NS5B and p7) but only included the “common” subtypes 1a, 1b, 2a and 3a.

## Competing interests

The authors declare that they have no competing interests.

## Authors’ contributions

NPS performed sub-genomic viral sequencing, sequence alignments, weblogos and epitope predictions. NPS also interpreted the data and drafted the manuscript. AJP participated in the design and concept and reviewed the manuscript. SMB conceived of the study, participated in the design, performed the population coverage calculations and had major input in the Discussion and Conclusions of the manuscript and also provided critical revision of the entire manuscript. All authors have read and approved the final manuscript.

## Supplementary Material

Additional file 1 **Figure S1. **An example of consensus Weblogos alignments for the NS3^1406-1415^ peptide for each of the 7 subtypes/genotypes studied. Percentage correspondence with the HCV consensus epitope 1407–1415. Average conservation was 65.17% (p = 0.1645), also shown in Table 2. Click here for file

Additional file 2 **Figure S2. **Epitope and population coverage in South African Blacks with original published epitopes, using IEDB. Click here for file

Additional file 3 **Figure S3. **Epitope and population coverage in South African Whites with original published epitopes, using IEDB. Click here for file

Additional file 4 **Figure S4. **Epitope and population coverage in South African Blacks with “best mix”, using IEDB. Click here for file

Additional file 5 **Figure S5. **Epitope and population coverage in South African Whites with “best mix”, using IEDB. Click here for file

Additional file 6 **Figure S6. **Epitope and population coverage in Caucasians (North American and Europe), using OptiTope. Click here for file

Additional file 7 **Figure S7. **Epitope and population coverage in Zulus (South Africa), using OptiTope. Click here for file

Additional file 8 **Figure S8. **A summary of the steps and results of the population coverage analyses, using the IEDB and OptiTope. Click here for file

## References

[B1] Choo QL Kuo G Weiner AJ Overby LR Bradley DW Houghton M Isolation of a cDNA clone derived from a blood-borne non-A, non-B viral hepatitis genome Science 1989 244 359 362 10.1126/science.2523562 2523562

[B2] Wakita T Pietschmann T Kato T Date T Miyamoto M Zhao Z Murthy K Habermann A Krausslich HG Mizokami M Bartenschlager R Liang TJ Production of infectious hepatitis C virus in tissue culture from a cloned viral genome Nat Med 2005 11 791 796 10.1038/nm1268 15951748 PMC2918402

[B3] Schulze ZurWiesch J Lauer GM Timm J Kuntzen T Neukamm M Berical A Jones AM Nolan BE Kasprowicz V McMahon C Wurcel A Lohse AW Lewis-Ximenez LL Chung RT Kim AY Allen TM Walker BD Longworth South Africa Immunologic evidence for lack of heterologous protection following resolution of HCV in patients with non-genotype 1 infection Blood 2007 110 1559 1569 10.1182/blood-2007-01-069583 17475911 PMC1975840

[B4] Klade CS Kubitschke A Stauber RE Meyer MF Zinke S Wiegand J Zauner W Aslan N Lehmann M Cornberg M Manns MP Reisner P Wedemeyer H Hepatitis C virus-specific T cell responses against conserved regions in recovered patients Vaccine 2009 27 3099 3108 10.1016/j.vaccine.2009.02.088 19428924

[B5] Bukh J A critical role for the chimpanzee model in the study of hepatitis C Hepatology 2004 39 1469 1475 10.1002/hep.20268 15185284

[B6] Ploss A Rice CM Towards a small animal model for hepatitis C EMBO Rep 2009 10 1220 1227 10.1038/embor.2009.223 19834510 PMC2775186

[B7] Dorner M Horwitz JA Robbins JB Barry WT Feng Q Mu K Jones CT Schoggins JW Catanese MT Burton DR Law M Rice CM Ploss A A genetically humanized mouse model for hepatitis C virus infection Nature 2011 474 208 211 10.1038/nature10168 21654804 PMC3159410

[B8] Grakoui A Shoukry NH Woollard DJ Han JH Hanson HL Ghrayeb J Murthy KK Rice CM Walker CM HCV persistence and immune evasion in the absence of memory T cell help Science 2003 302 659 662 10.1126/science.1088774 14576438

[B9] Wang JH Zheng X Ke X Dorak MT Shen J Boodram B O'Gorman M Beaman K Cotler SJ Hershow R Rong L Ethnic and geographical differences in HLA associations with the outcome of hepatitis C virus infection Virol J 2009 6 46 10.1186/1743-422X-6-46 19409091 PMC2679741

[B10] Neumann-Haefelin C Frick DN Wang JJ Pybus OG Salloum S Narula GS Eckart A Biezynski A Eiermann T Klenerman P Viazov S Roggendorf M Thimme R Reiser M Timm J Analysis of the evolutionary forces in an immunodominant CD8 epitope in hepatitis C virus at a population level J Virol 2008 82 3438 3451 10.1128/JVI.01700-07 18216107 PMC2268453

[B11] Sarobe P Lasarte JJ Garcia N Civeira MP Borras-Cuesta F Prieto J Characterization of T-cell responses against immunodominant epitopes from hepatitis C virus E2 and NS4a proteins J Viral Hepat 2006 13 47 55 10.1111/j.1365-2893.2005.00653.x 16364082

[B12] Satapathy SK Lingisetty CS Proper S Chaudhari S Williams S Equally poor outcomes to pegylated interferon-based therapy in African Americans and Hispanics with chronic hepatitis C infection J Clin Gastroenterol 2010 44 140 145 10.1097/MCG.0b013e3181ba9992 19826275

[B13] Paximadis M Mathebula TY Gentle NL Vardas E Colvin M Gray CM Tiemessen CT Puren A Human leukocyte antigen class I (A, B, C) and II (DRB1) diversity in the black and caucasian South African population Hum Immunol 2012 73 80 92 22074999 10.1016/j.humimm.2011.10.013

[B14] Statistics South Africa 2010 http://www.statssa.gov.za/PublicationsHTML/P03022010/html/P03022010.html

[B15] Prabdial-Sing N Puren AJ Mahlangu J Barrow P Bowyer SM Hepatitis C virus genotypes in two different patient cohorts in Johannesburg, South Africa Arch Virol 2008 153 2049 2058 10.1007/s00705-008-0227-2 18946631

[B16] Rosen HR Clinical practice. Chronic hepatitis C infection N Engl J Med 2011 364 25 2429 2438 10.1056/NEJMcp1006613 21696309

[B17] New allele Frequency Database 2003 http://www.allefrequencies.net

[B18] MacNamara A Kadolsky U Bangham CR Asquith B T-cell epitope prediction: rescaling can mask biological variation between MHC molecules PLoS Comput Biol 2009 5 3 e1000327 10.1371/journal.pcbi.1000327 19300484 PMC2650421

[B19] Lin HH Zhang GL Tongchusak S Reinherz EL Brusic V Evaluation of MHC-II peptide binding prediction servers: applications for vaccine research BMC Bioinformatics 2008 9 12 S22 10.1186/1471-2105-9-S12-S22 19091022 PMC2638162

[B20] Wertheimer AM Miner C Lewinsohn DM Sasaki AW Kaufman E Rosen HR Novel CD4+ and CD8+ T-cell determinants within the NS3 protein in subjects with spontaneously resolved HCV infection Hepatology 2003 37 577 589 10.1053/jhep.2003.50115 12601356

[B21] Wedemeyer H Schuller E Schlaphoff V Stauber RE Wiegand J Schiefke I Firbas C Jilma B Thursz M Zeuzem S Hofmann WP Hinrichsen H Tauber E Manns MP Klade CS Therapeutic vaccine IC41 as late add-on to standard treatment in patients with chronic hepatitis C Vaccine 2009 27 5142 5151 10.1016/j.vaccine.2009.06.027 19559112

[B22] Wei SH Yin W An QX Lei YF Hu XB Yang J Lu X Zhang H Xu ZK A novel hepatitis C virus vaccine approach using recombinant Bacillus Calmette-Guerin expressing multi-epitope antigen Arch Virol 2008 153 1021 1029 10.1007/s00705-008-0082-1 18421415

[B23] Memarnejadian A Roohvand F Arashkia A Rafati S Shokrgozar MA Polytope DNA vaccine development against hepatitis C virus: a streamlined approach from in silico design to in vitro and primary in vivo analyses in BALB/c mice Protein Pept Lett 2009 16 842 850 10.2174/092986609788681788 19601916

[B24] Cerny A McHutchison JG Pasquinelli C Brown ME Brothers MA Grabscheid B Fowler P Houghton M Chisari FV Cytotoxic T lymphocyte response to hepatitis C virus-derived peptides containing the HLA A2.1 binding motif J Clin Invest 1995 95 521 530 10.1172/JCI117694 7860734 PMC295505

[B25] Martin P Simon B Lone YC Chatel L Barry R Inchauspe G Fournillier A A vector-based minigene vaccine approach results in strong induction of T-cell responses specific of hepatitis C virus Vaccine 2008 26 2471 2481 10.1016/j.vaccine.2008.03.028 18423948

[B26] Lamonaca V Missale G Urbani S Pilli M Boni C Mori C Sette A Massari M Southwood S Bertoni R Valli A Fiaccadori F Ferrari C Conserved hepatitis C virus sequences are highly immunogenic for CD4(+) T cells: implications for vaccine development Hepatology 1999 30 1088 1098 10.1002/hep.510300435 10498664

[B27] Day CL Lauer GM Robbins GK McGovern B Wurcel AG Gandhi RT Chung RT Walker BD Broad specificity of virus-specific CD4+ T-helper-cell responses in resolved hepatitis C virus infection J Virol 2002 76 12584 12595 10.1128/JVI.76.24.12584-12595.2002 12438584 PMC136690

[B28] Diepolder HM Gerlach JT Zachoval R Hoffmann RM Jung MC Wierenga EA Scholz S Santantonio T Houghton M Southwood S Sette A Pape GR Immunodominant CD4+ T-cell epitope within nonstructural protein 3 in acute hepatitis C virus infection J Virol 1997 71 6011 6019 9223492 10.1128/jvi.71.8.6011-6019.1997 PMC191858

[B29] Schulze zur Wiesch J Lauer GM Day CL Kim AY Ouchi K Duncan JE Wurcel AG Timm J Jones AM Mothe B Allen TM McGovern B Lewis-Ximenez L Sidney J Sette A Chung RT Walker BD Broad repertoire of the CD4+ Th cell response in spontaneously controlled hepatitis C virus infection includes dominant and highly promiscuous epitopes J Immunol 2005 175 3603 3613 16148104 10.4049/jimmunol.175.6.3603

[B30] Sidney J Peters B Frahm N Brander C Sette A HLA class I supertypes: a revised and updated classification BMC Immunol 2008 9 1 10.1186/1471-2172-9-1 18211710 PMC2245908

[B31] Verbeeck J Maes P Lemey P Pybus OG Wollants E Song E Nevens F Fevery J Delport W Van der Merwe S Van Ranst M Investigating the origin and spread of hepatitis C virus genotype 5a J Virol 2006 80 4220 4226 10.1128/JVI.80.9.4220-4226.2006 16611881 PMC1472033

[B32] Henquell C Cartau C Abergel A Laurichesse H Regagnon C De Champs C Bailly JL Peigue-Lafeuille H High prevalence of hepatitis C virus type 5 in central France evidenced by a prospective study from 1996 to 2002 J Clin Microbiol 2004 42 3030 3035 10.1128/JCM.42.7.3030-3035.2004 15243055 PMC446309

[B33] Noppornpanth S Poovorawan Y Lien TX Smits SL Osterhaus AD Haagmans BL Complete genome analysis of hepatitis C virus subtypes 6t and 6u J Gen Virol 2008 89 1276 1281 10.1099/vir.0.83593-0 18420806

[B34] Farci P Alter HJ Govindarajan S Wong DC Engle R Lesniewski RR Mushahwar IK Desai SM Miller RH Ogata N Lack of protective immunity against reinfection with hepatitis C virus Science 1992 258 135 140 10.1126/science.1279801 1279801

[B35] Accapezzato D Fravolini F Casciaro MA Paroli M Hepatitis C flare due to superinfection by genotype 4 in an HCV genotype 1b chronic carrier Eur J Gastroenterol Hepatol 2002 14 879 881 10.1097/00042737-200208000-00012 12172410

[B36] Rauch A James I Pfafferott K Nolan D Klenerman P Cheng W Mollison L McCaughan G Shackel N Jeffrey GP Divergent adaptation of hepatitis C virus genotypes 1 and 3 to human leukocyte antigen-restricted immune pressure Hepatology 2009 50 1017 1029 10.1002/hep.23101 19670417

[B37] Fytili P Dalekos GN Schlaphoff V Suneetha PV Sarrazin C Zauner W Zachou K Berg T Manns MP Klade CS Cornberg M Wedemeyer H Cross-genotype-reactivity *of the immu*nodominant HCV CD8 T-cell epitope NS3-1073 Vaccine 2008 26 3818 3826 10.1016/j.vaccine.2008.05.045 18582999

[B38] Tong JC Tan TW Ranganathan S Methods and protocols for prediction of immunogenic epitopes Brief Bioinform 2007 8 96 108 17077136 10.1093/bib/bbl038

[B39] Rammensee H Bachmann J Emmerich NP Bachor OA Stevanovic S SYFPEITHI: database for MHC ligands and peptide motifs Immunogenetics 1999 50 213 219 10.1007/s002510050595 10602881

[B40] Lundegaard C Lund O Buus S Nielsen M Major histocompatibility complex class I binding predictions as a tool in epitope discovery Immunology 2010 130 3 309 318 10.1111/j.1365-2567.2010.03300.x 20518827 PMC2913210

[B41] Donnes P Elofsson A Prediction of MHC class I binding peptides, using SVMHC BMC Bioinformatics 2002 3 25 10.1186/1471-2105-3-25 12225620 PMC129981

[B42] Stranzl T Larsen MV Lundegaard C Nielsen M NetCTLpan: pan-specific MHC class I pathway epitope predictions Immunogenetics 2010 62 6 357 368 10.1007/s00251-010-0441-4 20379710 PMC2875469

[B43] McNamara LA He Y Yang Z Using epitope predictions to evaluate efficacy and population coverage of the Mtb72f vaccine for tuberculosis BMC Immunol 2010 11 18 10.1186/1471-2172-11-18 20353587 PMC2862017

[B44] Shehzadi A Ur Rehman S Idrees M Promiscuous prediction and conservancy analysis of CTL binding epitopes of HCV 3a viral proteome from Punjab Pakistan: an *in silico* approach Virol J 2011 8 55 10.1186/1743-422X-8-55 21303499 PMC3042956

[B45] Rai J Lok KI Mok CY Mann H Noor M Patel P Flower DR Immunoinformatic evaluation of multiple epitope ensembles as vaccine candidates: E coli 536 Bioinformation 2012 8 6 272 275 10.6026/97320630008272 22493535 PMC3321237

[B46] Dimitrov I Flower D Doytchinova I Improving *in silico* prediction of epitope vaccine candidates by union and intersection of single predictors World Journal of Vaccines 2011 1 2 15 22 10.4236/wjv.2011.12004

[B47] Ribeiro SP Rosa DS Fonseca SG Mairena EC Postol E Oliveira SC Guilherme L Kalil J Cunha-Neto E A vaccine encoding conserved promiscuous HIV CD4 epitopes induces broad T cell responses in mice transgenic to multiple common HLA class II molecules PLoS One 2010 5 6 e11072 10.1371/journal.pone.0011072 20552033 PMC2884037

[B48] Kim AY Kuntzen T Timm J Nolan BE Baca MA Reyor LL Berical AC Feller AJ Johnson KL Schulze Zur Wiesch J Spontaneous control of HCV is associated with expression of HLA-B 57 and preservation of targeted epitopes Gastroenterology 2011 140 2 686 696 e681 10.1053/j.gastro.2010.09.042 20875418 PMC3021586

[B49] Fitzmaurice K Petrovic D Ramamurthy N Simmons R Merani S Gaudieri S Sims S Dempsey E Freitas E Lea S Molecular footprints reveal the impact of the protective HLA-A*03 allele in hepatitis C virus infection Gut 2011 60 11 1563 1571 10.1136/gut.2010.228403 21551190 PMC3184218

[B50] Neumann-Haefelin C Kuntzen T Schmidt KN Sidney J Caillet-Saguy C Binder M Kersting MWK Power KA Ingber S Reyor LL Hills-Evans AYK Lauer GM Lohmann V Sette A Henn MR Thimme R Allen TM HLA-B27 selects for rare escape mutations that SignificantlyImpair Hepatitis C Virus replication and require compensatory mutations Hepatology 2011 54 4 1157 1166 10.1002/hep.24541 22006856 PMC3201753

[B51] Bowyer S Molecular characterization of the hepatitis B virus is South Africa. PhD thesis 2002 Johannesburg: University of the Witwatersrand, Department of Virology

[B52] Sette A Sidney J del Guercio MF Southwood S Ruppert J Dahlberg C Grey HM Kubo RT Peptide binding to the most frequent HLA-A class I alleles measured by quantitative molecular binding assays Mol Immunol 1994 31 813 822 10.1016/0161-5890(94)90019-1 8047072

[B53] Lang KA Yan J Draghia-Akli R Khan A Weiner DB Strong HCV NS3- and NS4A-specific cellular immune responses induced in mice and Rhesus macaques by a novel HCV genotype 1a/1b consensus DNA vaccine Vaccine 2008 26 6225 6231 10.1016/j.vaccine.2008.07.052 18692108 PMC4477808

[B54] Shin IT Tanaka Y Tateno Y Mizokami M Development and public release of a comprehensive hepatitis virus database Hepatol Res 2008 38 234 243 10.1111/j.1872-034X.2007.00262.x 17877727

[B55] Hall T BioEdit 1997 http://www.mbio.ncsu.edu

[B56] Choo QL Richman KH Han JH Berger K Lee C Dong C Gallegos C Coit D Medina-Selby R Barr PJ Genetic organization and diversity of the hepatitis C virus Proc Natl Acad Sci USA 1991 88 2451 2455 10.1073/pnas.88.6.2451 1848704 PMC51250

[B57] Crooks GE Hon G Chandonia JM Brenner SE WebLogo: a sequence logo generator Genome Res 2004 14 1188 1190 10.1101/gr.849004 15173120 PMC419797

[B58] Immune Epitope Database (IEDB) version 2.0 2010 http://tools.immuneepitope.org/main/jsp/menu.jsp

[B59] ProPred II 2001 http://www.imtech.res.in/raghava/propred/index.html

[B60] Vita R Zarebski L Greenbaum JA Emami H Hoof I Salimi N Damle R Sette A Peters B The immune epitope database 2.0 Nucleic Acids Res 2010 38 D854 62 10.1093/nar/gkp1004 19906713 PMC2808938

[B61] Nielsen M Lundegaard C Worning P Lauemoller SL Lamberth K Buus S Brunak S Lund O Reliable prediction of T-cell epitopes using neural networks with novel sequence representations Protein Sci 2003 12 1007 1017 10.1110/ps.0239403 12717023 PMC2323871

[B62] ProPred I 2003 http://www.imtech.res.in/raghava/propredI/index.html

[B63] Singh H Raghava GP ProPred: prediction of HLA-DR binding sites Bioinformatics 2001 17 1236 1237 10.1093/bioinformatics/17.12.1236 11751237

[B64] Toussaint NC Kohlbacher O OptiTope–a web server for the selection of an optimal set of peptides for epitope-based vaccines. Nucleic Acids Res 2009 37 W617 W622 10.1093/nar/gkp293 19420066 PMC2703925

