# Supplementary material for: Sequence-based in silico analysis of well studied Hepatitis C Virus epitopes and their variants in other genotypes (particularly genotype 5a) against South African human leukocyte antigen backgrounds
Source: BMC Immunol. 2012 Dec 10;13:67. doi: 10.1186/1471-2172-13-67 (PMC3552980; doi:10.1186/1471-2172-13-67)
Supplement: Additional file 1 — Figure S1. An example of consensus Weblogos alignments for the NS31406-1415 peptide for each of the 7 subtypes/genotypes studied. Percentage correspondence with the HCV consensus epitope 1407–1415. Average conservation was 65.17% (p = 0.1645), also shown in Table 2. [file 1471-2172-13-67-S1.pdf]

Subtype/Genotype	HCV Consensus: **LTSLGLNAV**	% Conservation
1a<br>1b	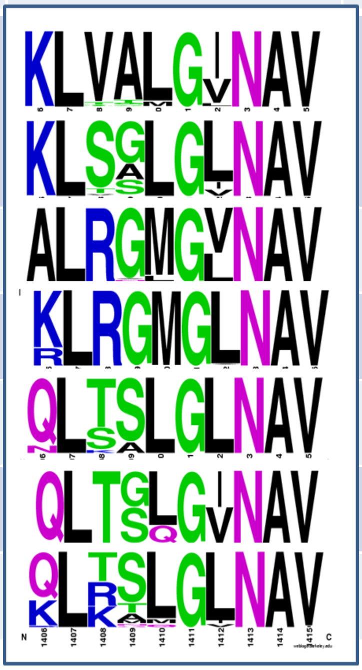	67
g2		56
g3		67
g4		78
5a		67
g6		56
