# Supplementary material for: Sequence-based in silico analysis of well studied Hepatitis C Virus epitopes and their variants in other genotypes (particularly genotype 5a) against South African human leukocyte antigen backgrounds
Source: BMC Immunol. 2012 Dec 10;13:67. doi: 10.1186/1471-2172-13-67 (PMC3552980; doi:10.1186/1471-2172-13-67)
Supplement: Additional file 2 — Figure S2. Epitope and population coverage in South African Blacks with original published epitopes, using IEDB. [file 1471-2172-13-67-S2.pdf]

Black-South African (u)

Epitope	Coverage Class I and II	HLA A*0201 (7.41)	HLA A*6802 (7.63)	HLA B*1503 (8.40)	HLA B*3501 (1.78)	HLA DRB1*0101 (1.32)	HLA DRB1*0102 (4.74)	HLA DRB1*0401 (2.63)	HLA DRB1*1301 (12.37)	HLA DRB1*1501 (2.37)	Total HLA hits
Epitope #1: cingvcwtv_1a	27.81%	+	+	-	-	-	-	-	-	-	2
Epitope #2: klvalgina_1a	14.26%	+	-	-	-	-	-	-	-	-	1
Epitope #3: llfnilggwv_1a_1b_4_5a	14.26%	+	-	-	-	-	-	-	-	-	1
Epitope #4: ilagygagv_1a_1b_5a	27.81%	+	+	-	-	-	-	-	-	-	2
Epitope #5: msyswtgal_1a_1b_4	31.16%	-	+	+	+	-	-	-	-	-	3
Epitope #6: glqdctmlv_1a	14.26%	+	-	-	-	-	-	-	-	-	1
Epitope #7: vyllprrgp_1_2_3_4_5_6	23.21%	-	-	-	-	-	-	-	+	-	1
Epitope #8: lvlnpsvaa_1_2_3_4_5_6	14.20%	-	-	-	-	-	+	+	-	-	2
Epitope #9: fnilggwva_1_4_5	11.75%	-	-	-	-	+	+	-	-	-	2
Epitope #10: NS5B2571_3_5	4.68%	-	-	-	-	-	-	-	-	+	1
Epitope set	65.85%	5	3	1	1	1	2	1	1	1	

\+ : restricted
\- : not restricted
shaded column : genotypic frequency of this allele is 0 (zero)
