# Supplementary material for: Sequence-based in silico analysis of well studied Hepatitis C Virus epitopes and their variants in other genotypes (particularly genotype 5a) against South African human leukocyte antigen backgrounds
Source: BMC Immunol. 2012 Dec 10;13:67. doi: 10.1186/1471-2172-13-67 (PMC3552980; doi:10.1186/1471-2172-13-67)
Supplement: Additional file 3 — Figure S3. Epitope and population coverage in South African Whites with original published epitopes, using IEDB. [file 1471-2172-13-67-S3.pdf]

White-South African (u)

Epitope	Coverage Class I and II	HLA A*0201 (25.34)	HLA A*6802 (0.97)	HLA B*1503 (0.51)	HLA B*3501 (6.15)	HLA DRB1*0101 (7.45)	HLA DRB1*0102 (1.06)	HLA DRB1*0401 (11.17)	HLA DRB1*1301 (6.38)	HLA DRB1*1501 (11.17)	Total HLA hits
		HLA allele (genotypic frequency (%))									
Epitope #1: cingvcwtv_1a	45.71%	+	+	-	-	-	-	-	-	-	2
Epitope #2: klvalgina_1a	44.26%	+	-	-	-	-	-	-	-	-	1
Epitope #3: llfnilggwv_1a_1b_4_5a	44.26%	+	-	-	-	-	-	-	-	-	1
Epitope #4: ilagygagv_1a_1b_5a	45.71%	+	+	-	-	-	-	-	-	-	2
Epitope #5: msyswtgal_1a_1b_4	14.57%	-	+	+	+	-	-	-	-	-	3
Epitope #6: glqdctmlv_1a	44.26%	+	-	-	-	-	-	-	-	-	1
Epitope #7: vyllprrgp_1_2_3_4_5_6	12.35%	-	-	-	-	-	-	-	+	-	1
Epitope #8: lvlnpsvaa_1_2_3_4_5_6	22.96%	-	-	-	-	-	+	+	-	-	2
Epitope #9: fnilggwva_1_4_5	16.30%	-	-	-	-	+	+	-	-	-	2
Epitope #10: NS5B2571_3_5	21.09%	-	-	-	-	-	-	-	-	+	1
Epitope set	81.36%	5	3	1	1	1	2	1	1	1	

+ : restricted
- : not restricted
shaded column : genotypic frequency of this allele is 0 (zero)
