# Supplementary material for: Sequence-based in silico analysis of well studied Hepatitis C Virus epitopes and their variants in other genotypes (particularly genotype 5a) against South African human leukocyte antigen backgrounds
Source: BMC Immunol. 2012 Dec 10;13:67. doi: 10.1186/1471-2172-13-67 (PMC3552980; doi:10.1186/1471-2172-13-67)
Supplement: Additional file 4 — Figure S4. Epitope and population coverage in South African Blacks with “best mix”, using IEDB. [file 1471-2172-13-67-S4.pdf]

# Black-South African (u)

| Epitope | Coverage Class I and II | HLA A*0201 (7.41) | HLA A*0205 (6.06) | HLA A*3001 (9.42) | HLA A*6802 (7.63) | HLA B*1503 (8.40) | HLA B*3501 (1.78) | HLA B*5801 (8.14) | HLA DRB1*0101 (1.32) | HLA DRB1*0102 (4.74) | HLA DRB1*0301 (7.63) | HLA DRB1*0401 (2.63) | HLA DRB1*0701 (7.11) | HLA DRB1*1101 (11.58) | HLA DRB1*1301 (12.37) | HLA DRB1*1501 (2.37) | Total HLA hits |
|---|---|---|---|---|---|---|---|---|---|---|---|---|---|---|---|---|---|
| | | HLA allele (genotypic frequency (%)) | | | | | | | | | | | | | | | |
| Epitope #1: cingvlwtv_1a_ | 25.11% | + | + | - | - | - | - | - | - | - | - | - | - | - | - | - | 2 |
| Epitope #2: cingvmwtl_1b_ | 25.11% | + | + | - | - | - | - | - | - | - | - | - | - | - | - | - | 2 |
| Epitope #3: llfnilggwv_1a,1b,4,5a_ | 27.65% | + | - | - | - | - | - | + | - | - | - | - | - | - | - | - | 2 |
| Epitope #4: msyswtgal_1a,1b,4_ | 39.89% | - | + | - | + | + | + | - | - | - | - | - | - | - | - | - | 4 |
| Epitope #5: mysytwtgal_5a_ | 39.89% | - | + | - | + | + | + | - | - | - | - | - | - | - | - | - | 4 |
| Epitope #6: klrdctllv_5a_ | 40.53% | + | + | + | - | - | - | - | - | - | - | - | - | - | - | - | 3 |
| Epitope #7: NS3_1252_1,2,3,4,5,6_ | 74.75% | - | - | - | - | - | - | - | + | + | + | + | + | + | + | + | 8 |
| Epitope #8: NS4_1809_1,4,5_ | 63.39% | - | - | - | - | - | - | - | + | + | - | - | + | + | + | + | 6 |
| Epitope #9: Core_1,2,4,5,6_ | 23.21% | - | - | - | - | - | - | - | - | - | - | - | - | - | + | - | 1 |
| Epitope set | 91.87% | 4 | 5 | 1 | 2 | 2 | 2 | 1 | 2 | 2 | 1 | 1 | 2 | 2 | 3 | 2 | |

+ : restricted
- : not restricted
shaded column : genotypic frequency of this allele is 0 (zero)
