# Supplementary material for: Sequence-based in silico analysis of well studied Hepatitis C Virus epitopes and their variants in other genotypes (particularly genotype 5a) against South African human leukocyte antigen backgrounds
Source: BMC Immunol. 2012 Dec 10;13:67. doi: 10.1186/1471-2172-13-67 (PMC3552980; doi:10.1186/1471-2172-13-67)
Supplement: Additional file 5 — Figure S5. Epitope and population coverage in South African Whites with “best mix”, using IEDB. [file 1471-2172-13-67-S5.pdf]

White-South African (u)

Epitope	Coverage	HLA allele (genotypic frequency (%))															Total HLA hits
	Class I and II	HLA A*0201 (25.34)	HLA A*0205 (0.49)	HLA A*3001 (1.46)	HLA A*6802 (0.97)	HLA B*1503 (0.51)	HLA B*3501 (6.15)	HLA B*5801 (2.56)	HLA DRB1*0101 (7.45)	HLA DRB1*0102 (1.06)	HLA DRB1*0301 (12.23)	HLA DRB1*0401 (11.17)	HLA DRB1*0701 (9.57)	HLA DRB1*1101 (5.85)	HLA DRB1*1301 (6.38)	HLA DRB1*1501 (11.17)	
Epitope #1: cingvlwtv_1a_	44.99%	+	+	-	-	-	-	-	-	-	-	-	-	-	-	-	2
Epitope #2: cingvmwtl_1b_	44.99%	+	+	-	-	-	-	-	-	-	-	-	-	-	-	-	2
Epitope #3: llfnilggwv_1a,1b,4,5a_	47.08%	+	-	-	-	-	-	+	-	-	-	-	-	-	-	-	2
Epitope #4: msyswtgal_1a,1b,4_	15.41%	-	+	-	+	+	+	-	-	-	-	-	-	-	-	-	4
Epitope #5: mysytwtgal_5a_	15.41%	-	+	-	+	+	+	-	-	-	-	-	-	-	-	-	4
Epitope #6: klrdctllv_5a_	47.13%	+	+	+	-	-	-	-	-	-	-	-	-	-	-	-	3
Epitope #7: NS3_1252_1,2,3,4,5,6_	87.67%	-	-	-	-	-	-	-	+	+	+	+	+	+	+	+	8
Epitope #8: NS4_1809_1,4,5_	65.75%	-	-	-	-	-	-	-	+	+	-	-	+	+	+	+	6
Epitope #9: Core_1,2,4,5,6_	12.35%	-	-	-	-	-	-	-	-	-	-	-	-	-	+	-	1
Epitope set	94.77%	4	5	1	2	2	2	1	2	2	1	1	2	2	3	2	

+ : restricted
- : not restricted
shaded column : genotypic frequency of this allele is 0 (zero)
