# Supplementary material for: Sequence-based in silico analysis of well studied Hepatitis C Virus epitopes and their variants in other genotypes (particularly genotype 5a) against South African human leukocyte antigen backgrounds
Source: BMC Immunol. 2012 Dec 10;13:67. doi: 10.1186/1471-2172-13-67 (PMC3552980; doi:10.1186/1471-2172-13-67)
Supplement: Additional file 6 — Figure S6. Epitope and population coverage in Caucasians (North American and Europe), using OptiTope. [file 1471-2172-13-67-S6.pdf]

OptiTope

Target Antigens > Target Population > Constraints > Results

In this final step the optimization problem is summarized and the results are displayed. Hel?

Input summary

Number of candidate epitopes:	2930
Number of target alleles:	19
Number of antigens:	10
Prediction method:	BIMAS

Constraints

Maximum number of epitopes to select	=	10
Epitope conservation	≥	20.0 %
Covered alleles	≥	10
Covered antigens	≥	5

Results

Click here for more information

Selected epitopes:	10	
Covered alleles:	10 of 19	
Covered antigens:	5 of 10	
Locus coverage:		
	A	54.01 %
	B	19.87 %
	Cw	35.11 %
Population coverage:		94.28 %

Epitope	Fraction of overall immunogenicity	Covered alleles	Covered antigens
VLTDFKTWL	0.16	A*0201	ns5a
KLLPRLPGV	0.14	A*0201	ns5a
QLDLSGWFV	0.11	A*0201	ns5b
YLYGIGSAV	0.11	A*0201	e2
KLQDCTMLV	0.11	A*0201	ns5b
LLSTTEWQV	0.1	A*0201	e2
FLATCVNGV	0.1	A*0201	ns3
ALYDVVSTL	0.1	A*0201 A*0301	ns5b
GPTPLLYRL	0.05	B*0702 B*3501 Cw*0401 Cw*0602 Cw*0702	ns3
GVVCAAILR	0.03	A*1101 A*3101 A*6801	ns4b

☐ Show detailed results.

☐ Export results.

<< previous
