# Supplementary material for: Sequence-based in silico analysis of well studied Hepatitis C Virus epitopes and their variants in other genotypes (particularly genotype 5a) against South African human leukocyte antigen backgrounds
Source: BMC Immunol. 2012 Dec 10;13:67. doi: 10.1186/1471-2172-13-67 (PMC3552980; doi:10.1186/1471-2172-13-67)
Supplement: Additional file 7 — Figure S7. Epitope and population coverage in Zulus (South Africa), using OptiTope. [file 1471-2172-13-67-S7.pdf]

OptiTope

Target Antigens > Target Population > Constraints > Results

In this final step the optimization problem is summarized and the results are displayed. Hel?

Input summary

Number of candidate epitopes:	2930
Number of target alleles:	16
Number of antigens:	10
Prediction method:	BIMAS

Constraints

Maximum number of epitopes to select	=	10
Epitope conservation	≥	20.0 %
Covered alleles	≥	8
Covered antigens	≥	5

Results

Click here for more information

Selected epitopes:	10	
Covered alleles:	10 of 16	
Covered antigens:	6 of 10	
Locus coverage:		
	A	15.46 %
	B	21.4 %
	Cw	25.0 %
Population coverage:		75.16 %

Epitope	Fraction of overall immunogenicity	Covered alleles	Covered antigens
EYVLLLFLL	0.14	A*2402 Cw*0401 Cw*0602	e2
HYKVFLARL	0.13	A*2402 Cw*0401 Cw*0602	ns2
HSKKKCDEL	0.1	B*0801	ns3
EARQAIRSL	0.1	B*0702 B*0801	ns5b
RYAPACKPL	0.1	A*2402 Cw*0401	ns5a
VEVTRVGDF	0.09	B*4403	ns5a
EVDGVRLHR	0.09	A*0101 A*6801	ns5a
QYLAGLSTL	0.09	A*2402 Cw*0401	ns4b
GPTPLLYRL	0.08	B*0702 B*3501 Cw*0401 Cw*0602 Cw*0702	ns3
MYTNVDQDL	0.08	A*2402 Cw*0401	ns3

☐ Show detailed results.

☐ Export results.

<< previous
