# Supplementary material for: Sequence-based in silico analysis of well studied Hepatitis C Virus epitopes and their variants in other genotypes (particularly genotype 5a) against South African human leukocyte antigen backgrounds
Source: BMC Immunol. 2012 Dec 10;13:67. doi: 10.1186/1471-2172-13-67 (PMC3552980; doi:10.1186/1471-2172-13-67)

IEDB Population coverage

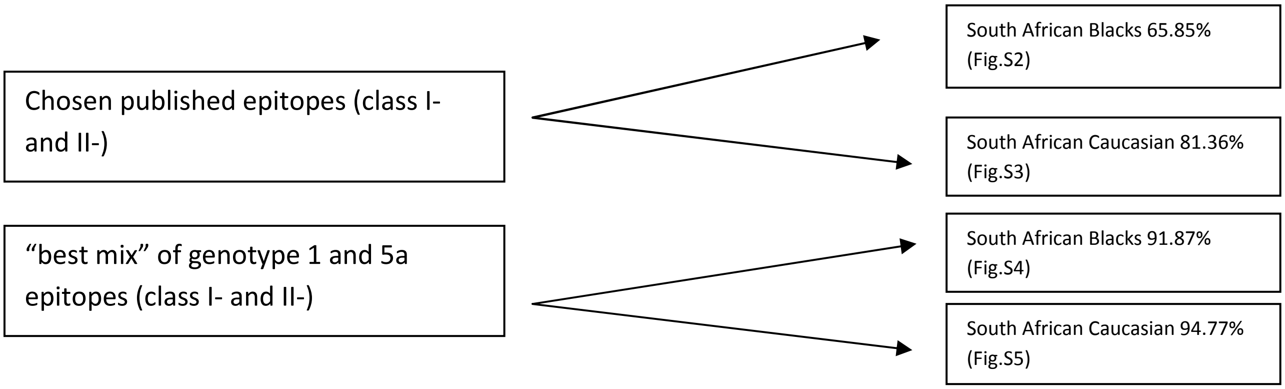

OptiTope Population coverage

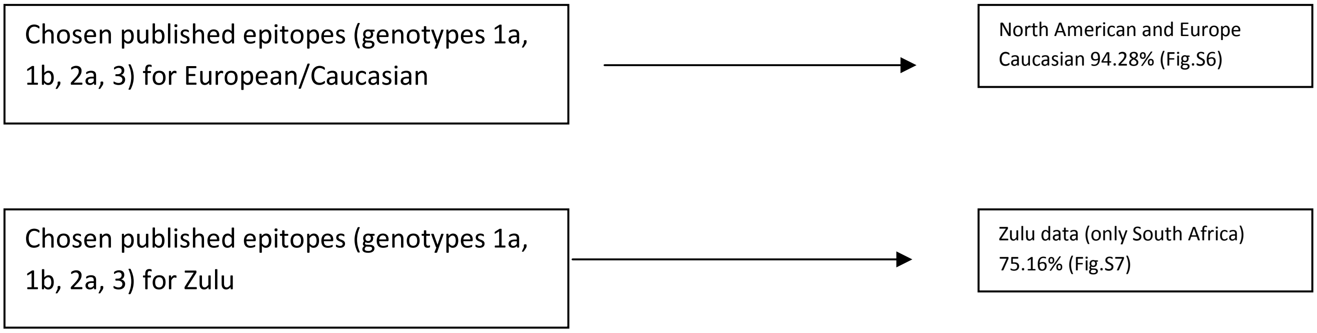

OptiTope epitopes and placed in IEDB Population coverage tool

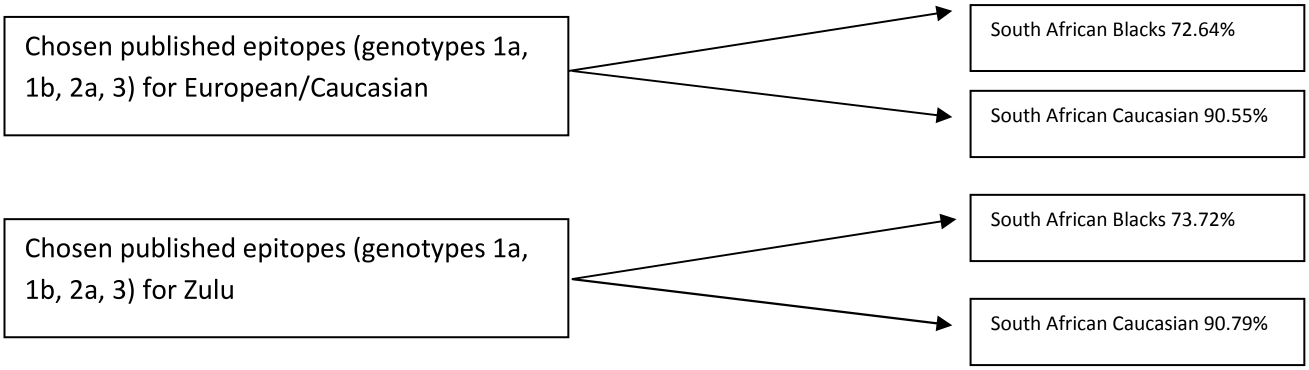

Supplement: Additional file 8 — Figure S8. A summary of the steps and results of the population coverage analyses, using the IEDB and OptiTope. [file 1471-2172-13-67-S8.pdf]
